# Early pregnancy maternal progesterone administration alters pituitary and testis function and steroid profile in male fetuses

**DOI:** 10.1038/s41598-020-78976-x

**Published:** 2020-12-14

**Authors:** Katarzyna J. Siemienowicz, Yili Wang, Magda Marečková, Junko Nio-Kobayashi, Paul A. Fowler, Mick T. Rae, W. Colin Duncan

**Affiliations:** 1 MRC Centre for Reproductive Health, The University of Edinburgh, Edinburgh, EH16 4TJ UK; 2 School of Applied Sciences, Edinburgh Napier University, Edinburgh, EH11 4BN UK; 3 Laboratory of Histology and Cytology, Hokkaido University Graduate School of Medicine, Sapporo, 060-9368 Japan; 4 Institute of Medical Sciences, School of Medicine, Medical Sciences and Nutrition, University of Aberdeen, Aberdeen, AB25 2ZD UK

**Keywords:** Developmental biology, Diseases, Endocrinology, Medical research

## Abstract

Maternal exposure to increased steroid hormones, including estrogens, androgens or glucocorticoids during pregnancy results in chronic conditions in offspring that manifest in adulthood. Little is known about effects of progesterone administration in early pregnancy on fetal development. We hypothesised that maternal early pregnancy progesterone supplementation would increase fetal progesterone, affect progesterone target tissues in the developing fetal reproductive system and be metabolised to other bioactive steroids in the fetus. We investigated the effects of progesterone treatment during early pregnancy on maternal and fetal plasma progesterone concentrations, transcript abundance in the fetal pituitary and testes and circulating steroids, at day 75 gestation, using a clinically realistic ovine model. Endogenous progesterone concentrations were lower in male than female fetuses. Maternal progesterone administration increased male, but not female, fetal progesterone concentrations, also increasing circulating 11-dehydrocorticosterone in male fetuses. Maternal progesterone administration altered fetal pituitary and testicular function in ovine male fetuses. This suggests that there may be fetal sex specific effects of the use of progesterone in early pregnancy, and highlights that progesterone supplementation should be used only when there is clear evidence of efficacy and for as limited time as necessary.

## Introduction

Fetal exposure to sex steroids has critical roles in sexual differentiation and the programming of health and disease in later life^1^. Exposure to endocrine disrupting compounds is linked to disease development in offspring^2^. Endocrine disruption classically involves alteration of steroid signalling during fetal development through maternal exposure^3^. Alteration in fetal hormone exposure can have lifelong effects.

Early pregnancy supplementation with the potent estrogen diethylstilbestrol (DES), to prevent miscarriage, between 1938 and 1971 resulted in a generation of children with disparate disorders as a consequence. Increased gestational exposure to estrogens is associated with developmental defects of the reproductive system in both male and female offspring, tumour development and subfertility in adult life^4^. Likewise, oral hormone pregnancy tests (HPTs), such as Primodos, containing ethinylestradiol and high doses of synthetic progesterone, available from 1958 to 1978, were first implicated in 1967 as a possible cause of birth defects^5,6^. Recent meta-analysis concluded that HPTs were associated with a 40% increased risk of congenital malformations^7^. In addition, exposure to excess androgen concentrations in utero is associated with a polycystic ovary phenotype in female offspring in both animal models and humans^8–11^. Both male and female offspring exposed to excess androgens prenatally show insulin resistance and dyslipidaemia in adulthood^12,13^. Animal models and human studies show that increased exposure to glucocorticoids during development is associated with insulin resistance and metabolic dysfunction in adulthood^14,15^.

Progesterone supplementation during early gestation is common during assisted reproduction^16^ and it is frequently sought and prescribed as a treatment for increased risk of miscarriage up to 16 weeks’ human gestation^17,18^. There is limited information concerning whether progesterone elevation during pregnancy has programming effects, like those seen with estrogen, androgens and glucocorticoids. The “Goldilocks” concept of steroid exposure during development suggests that too little or too much hormone is detrimental and that it needs to be just right^19^.

We hypothesised that: (1) maternal progesterone supplementation in early pregnancy would increase fetal progesterone, (2) an increase in fetal progesterone would alter the function of developing reproductive tissues, and (3) increased progesterone would be metabolised to other bioactive steroids in the fetus. We used a clinically realistic ovine model where we studied fetuses at day 75 of gestation, with the developmental stage approximately equivalent to the 15-week human fetus^20^. We investigated the effects of early-pregnancy maternal progesterone treatment on maternal and fetal plasma progesterone concentrations, transcript abundance in the fetal liver, pituitary and testes and circulating steroids, where progesterone is a precursor molecule.

## Results

### Fetal progesterone concentrations at D75 of gestation and effect of maternal progesterone administration

**Figure 1:**
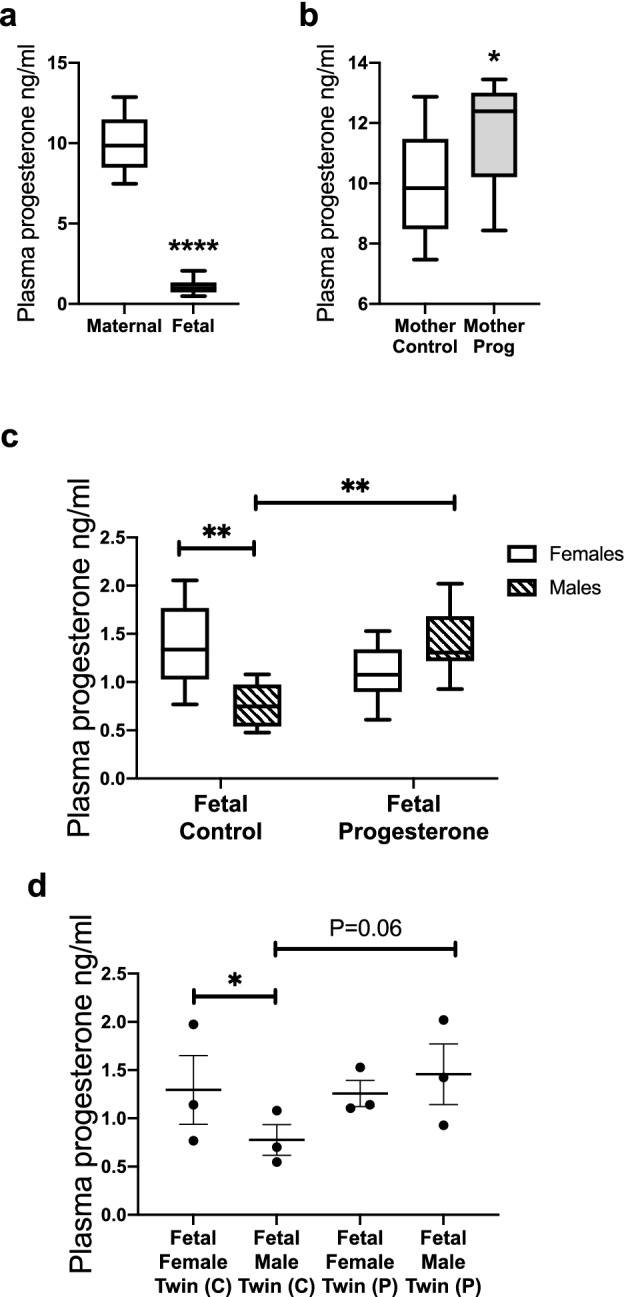
Fetal progesterone concentrations at d75 gestation. (**a**) Progesterone concentrations from the control ewes (n = 10) and their fetuses (n = 20). (**b**) Maternal progesterone was increased after the progesterone treatment (C = 10; P = 10). (**c**) Fetal female controls (n = 10) had higher progesterone concentration than fetal male controls (n = 10). Maternal progesterone administration had no effect on female fetuses (n = 13) but increased male fetal progesterone concentration (n = 7). (**d**) The difference in progesterone concentration was present in control male and female co-twins (n = 3). The sexually dimorphic effect of maternal progesterone treatment was seen in male/female co-twin pairs (C = 3; P = 3). Box plot whiskers are lowest and highest observed values, box is the upper and lower quartile, with median represented by line in box. Unpaired, two-tailed Student’s *t* test was used for comparing means of two treatment groups with equal variances accepting *P* < 0.05 as significant. Maternal progesterone was analysed using unpaired, one-tailed Student’s *t* test. The effect of progesterone treatment on female and male fetuses was analysed using two-way ANOVA with Tukey post hoc test. Co-twin fetal serum progesterone level between male and female was analysed using a paired two-tailed Student’s *t* test and between progesterone and control a one-tail unpaired Student’s *t* test (*P < 0.05; **P < 0.01; ***P < 0.001; ****P < 0.0001).

Progesterone is present in the fetal circulation at a concentration around 10 times lower than in the maternal circulation (Fig. 1a; *P* < 0.0001). Gestational progesterone treatment increased maternal progesterone concentrations (Fig. 1b; *P* < 0.05). Circulating progesterone concentrations were lower in male fetuses (Fig. 1c; *P* < 0.01) than female fetuses and this relationship was maintained in male/female co-twins (Fig. 1d; *P* < 0.05). Circulating fetal progesterone concentrations show sexual dimorphism.

There was no effect of exogenous progesterone administration on circulating progesterone concentrations in female fetuses, however, in male fetuses maternal progesterone administration increased circulating progesterone concentrations to female levels (Fig. 1c; *P* < 0.01). This differential effect was also observed in male/female co-twins (Fig. 1d; *P* = 0.06), suggesting little endocrine exchange between twins. Male fetal progesterone increases in response to maternal progesterone administration.

### Sexual dimorphism in fetal hepatic progesterone metabolism

**Figure 2:**
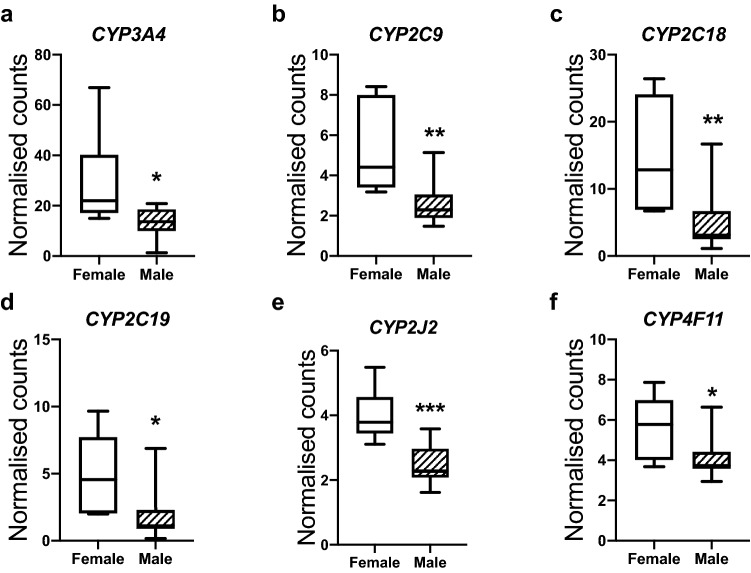
Sexually dimorphic expression of genes involved in progesterone and xenobiotic metabolism in fetal female (n = 6) and male (n = 11) livers at day 90 of gestation. Data represent RNAseq normalised gene counts. These data resulted from a separate study involving detailed hepatic analysis. Box plot whiskers are lowest and highest observed values, box is the upper and lower quartile, with median represented by line in box. Unpaired, two-tailed Student’s *t* test was used for comparing means of two treatment groups with equal variances accepting *P* < 0.05 as significant (*P < 0.05, **P < 0.01).

We conducted an in silico analysis of the ovine fetal liver transcriptome using an existing dataset (see^13^). This dataset, from a separate study, consisted of hepatic RNAseq analysis performed on control fetal female and male livers at a slightly later gestation (gestational D90). Expression of hepatic progesterone metabolising enzymes *CYP3A4*, *CYP2C9*, *CYP2C19*^21,22^ and xenobiotic metabolising enzymes *CYP2C18, CYP2J2* and *CYP4F11*^23,24^ were decreased in fetal males as compared with fetal females (Fig. 2a–f; *P* < 0.05–0.001). Expression of progesterone metabolising enzymes in the fetal liver is sexually dimorphic.

### Sites of progesterone action in the male fetus at D75 of gestation

**Figure 3:**
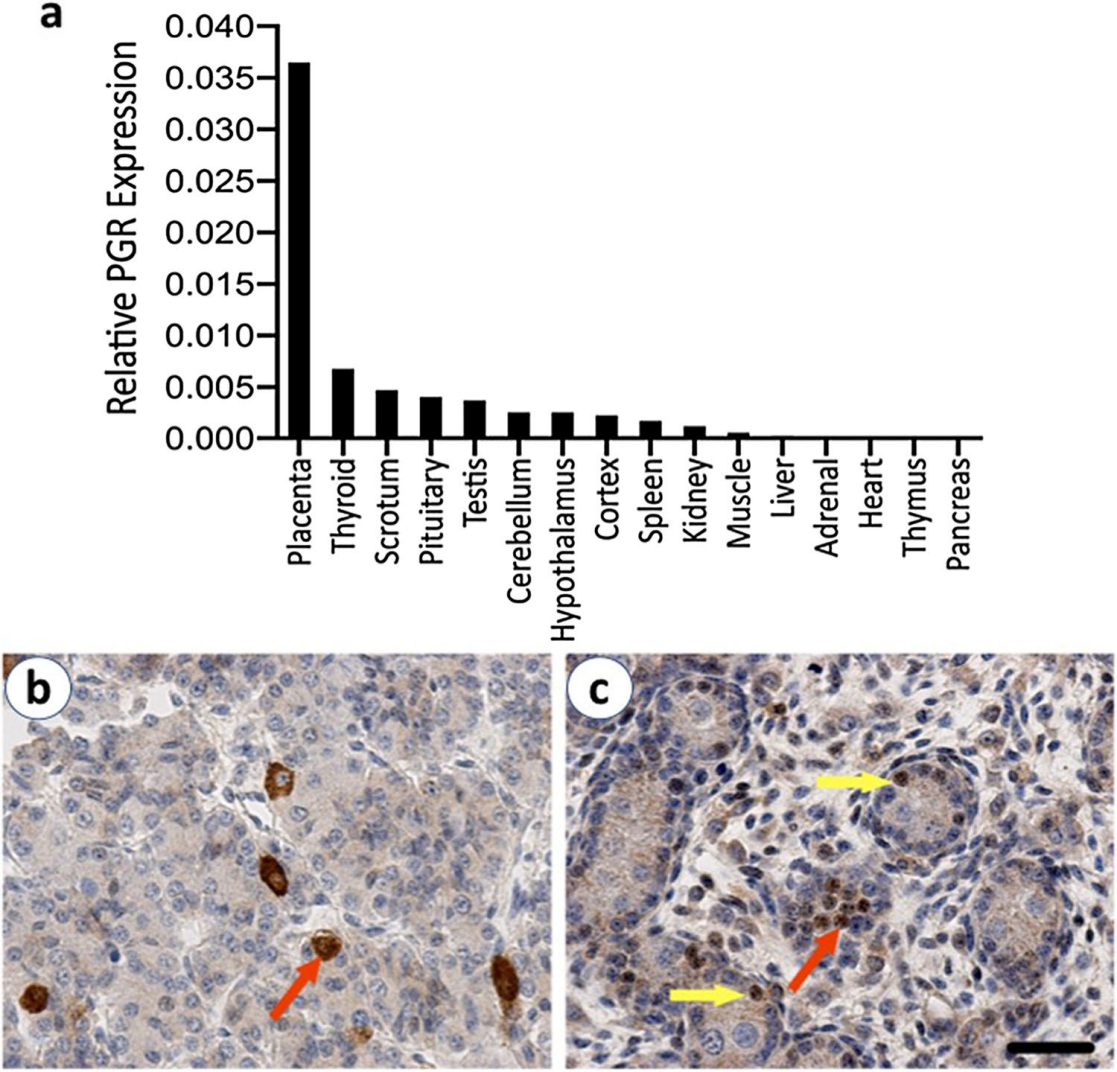
The expression of progesterone receptors in the male fetus at d75 gestation. (**a**) *PGR* transcript abundance in various fetal tissues in a control male fetus. (**b**) Immunolocalisation of PGR (brown–red arrow) to individual cells in a representative control male fetal pituitary at d75 gestation. (**c**) Immunolocalisation of PGR (brown) to Sertoli cells (yellow arrows) and fetal Leydig cells (red arrow) in a representative control fetal testis at d75 gestation. Scale bar = 50 µm.

The transcript abundance of progesterone receptor was addressed in multiple tissues in the male fetus (Fig. 3a). The placenta itself expresses progesterone receptors as does the fetal thyroid. We further examined progesterone receptors in the two reproductive tissues of interest; the pituitary gland and testis. PGR was immunolocalised to individual cells in the fetal pituitary (Fig. 3b) as well as Leydig and Sertoli cells in the developing testis (Fig. 3c). Progesterone therefore has the potential to act on the developing hypothalamo-pituitary-testis axis in the male fetus.

### Effect on progesterone on the fetal pituitary at D75 of gestation

**Figure 4:**
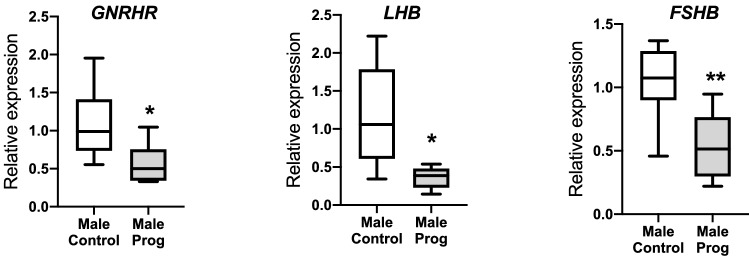
The effect of maternal progesterone on pituitary gene expression in male fetuses at d75 gestation compared to vehicle controls. Maternal progesterone administration decreased expression of *GNRHR*, *FSHB* and *LHB* in fetal males (C = 10; P = 7). Box plot whiskers are lowest and highest observed values, box is the upper and lower quartile, with median represented by line in box. Unpaired, two-tailed Student’s *t* test was used for comparing means of two treatment groups with equal variances accepting *P* < 0.05 as significant (*P < 0.05, **P < 0.01).

**Figure 5:**
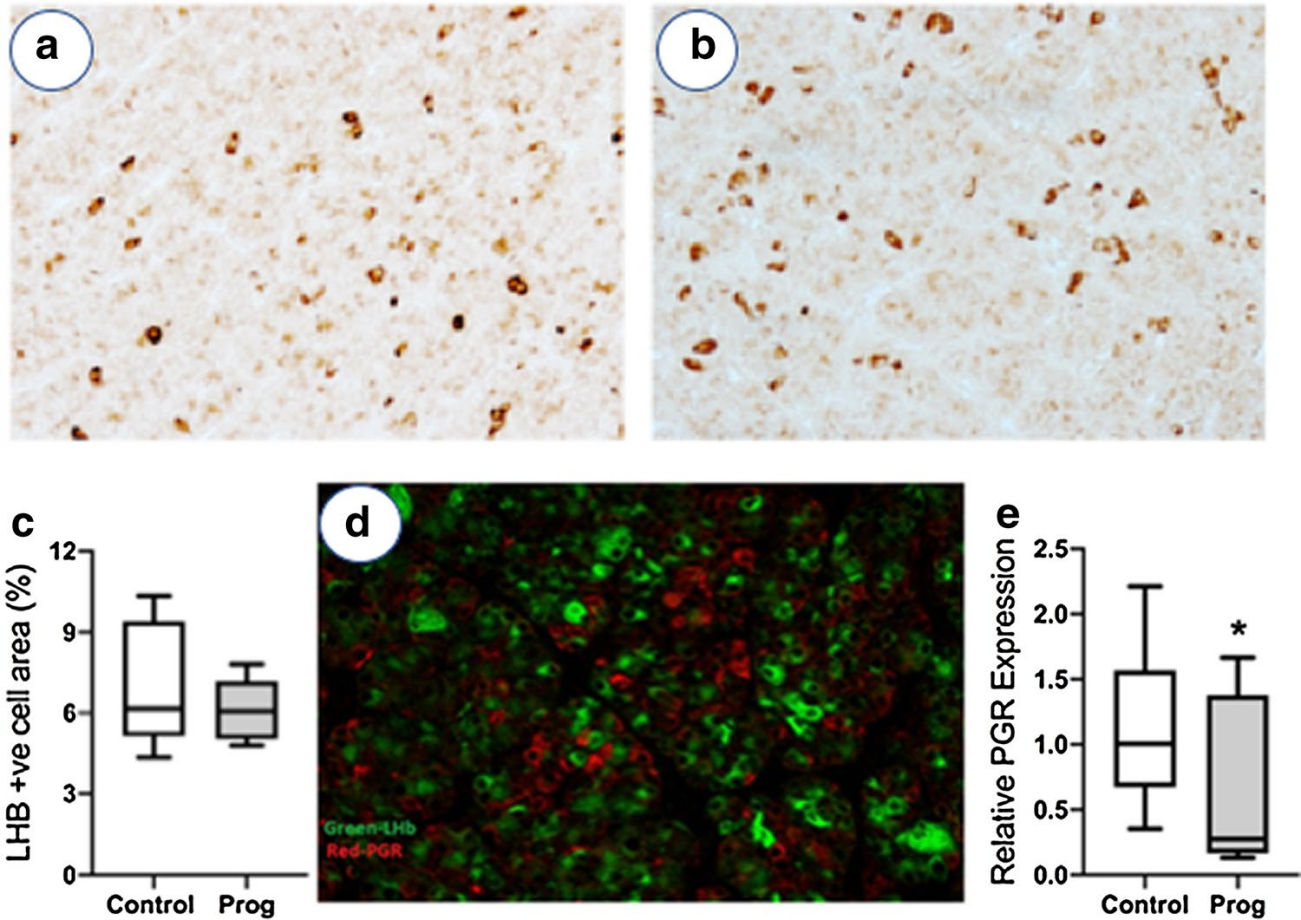
Assessment of gonadotrophs in the fetal pituitary at d75 gestation. Representative immunostaining for LHB (brown) in (**a**) the control fetal male pituitary and (**b**) the fetal male pituitary after maternal progesterone administration. (**c**) There was no difference in the area of gonadotroph staining (LHB) in the male fetal pituitary after maternal treatment with vehicle control (Control) or progesterone (Prog) (C = 10; P = 7). (**d**) Representative dual immunostaining of a control male fetal pituitary showing LHB (green) and PGR (red) showing that the gonadotrophs do not express PGR. (**e**) Maternal progesterone administration (Prog) decreased *PGR* expression in the male fetal pituitary (C = 10; P = 7). Box plot whiskers are lowest and highest observed values, box is the upper and lower quartile, with median represented by line in box. Unpaired, two-tailed Student’s *t* test was used for comparing means of two treatment groups with equal variances accepting *P* < 0.05 as significant (*P < 0.05).

In the male [but not female (Supplementary Fig. 1)] fetal pituitary, exogenous progesterone treatment decreased *GNRHR* expression (*P* < 0.05) and substantially reduced *FSHB* (*P* < 0.01) and *LHB* (*P* < 0.05) transcript abundance (Fig. 4). Progesterone treatment also decreased *PGR* expression in fetal male pituitary (Fig. 5e; *P* < 0.05). In addition, the degree of suppression of *LHB*, *FSHB* and *PGR* correlated with circulating progesterone concentrations (Supplementary Table 1). In order to determine if this was a reduction in the number of gonadotrophs or their function, gonadotroph cells were identified by immunohistochemistry for LHB (Fig. 5a,b). There was no difference in gonadotroph area in the male fetal pituitary after maternal progesterone administration (Fig. 5c). PGR did not co-localise with LHB in the fetal pituitary (Fig. 5d). Increased fetal progesterone has effects on the male fetal pituitary gonadotroph function that may not be a direct effect on gonadotrophs.

### Effect of progesterone on the fetal testis at D75 of gestation

**Figure 6:**
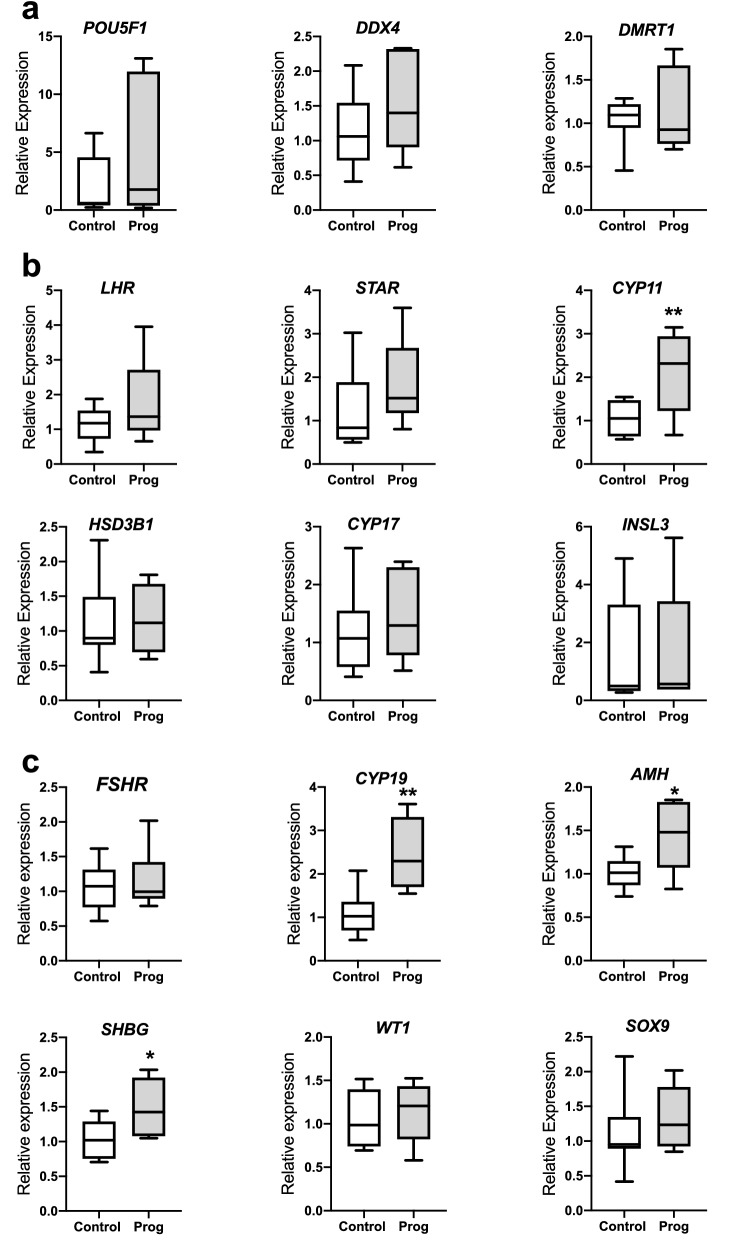
The effect of maternal progesterone administration (Prog) on gene expression in the fetal testis at d75 gestation (Control = 10; Prog = 7). (**a**) There was no difference in *POU5F1*, *VASA* and *DMRT1* that are key genes expressed in germ cells (**b**) There was no difference in the expression of *LHR*, *STAR*, *HSD3B1*, *CYP17* and *INSL3* that are key genes expressed in Leydig cells. However, expression of *CYP11* was increased in Leydig cells after maternal progesterone administration. (**c**) There was no significant change of expression of *FSHR*, *WT1*, *SOX9* that are primarily expressed in Sertoli cells. However other key genes (*CYP19*, *AMH*, *SHBG*) expressed in Sertoli cells were increased after maternal progesterone administration. Box plot whiskers are lowest and highest observed values, box is the upper and lower quartile, with median represented by line in box. Unpaired, two-tailed Student’s *t* test was used for comparing means of two treatment groups with equal variances accepting *P* < 0.05 as significant (*P < 0.05, **P < 0.01).

The effect of maternal administration of progesterone on testicular development was assessed by examining expression of genes that are primarily expressed in the different cell types within the fetal testes. There were no significant differences in genes associated with germ cell function (Fig. 6a). However, maternal progesterone exposure increased the expression of *CYP11* in Leydig cells and three genes (*CYP19*, *AMH* and *SHBG*) involved in Sertoli cell function (Fig. 6b,c respectively; *P* < 0.05–0.01). Further, there was a significant positive correlation between progesterone concentration and expression of several genes in fetal testes (Supplementary Table 1).

### Effects of progesterone on the male fetal steroid profile at D75 of gestation

**Figure 7:**
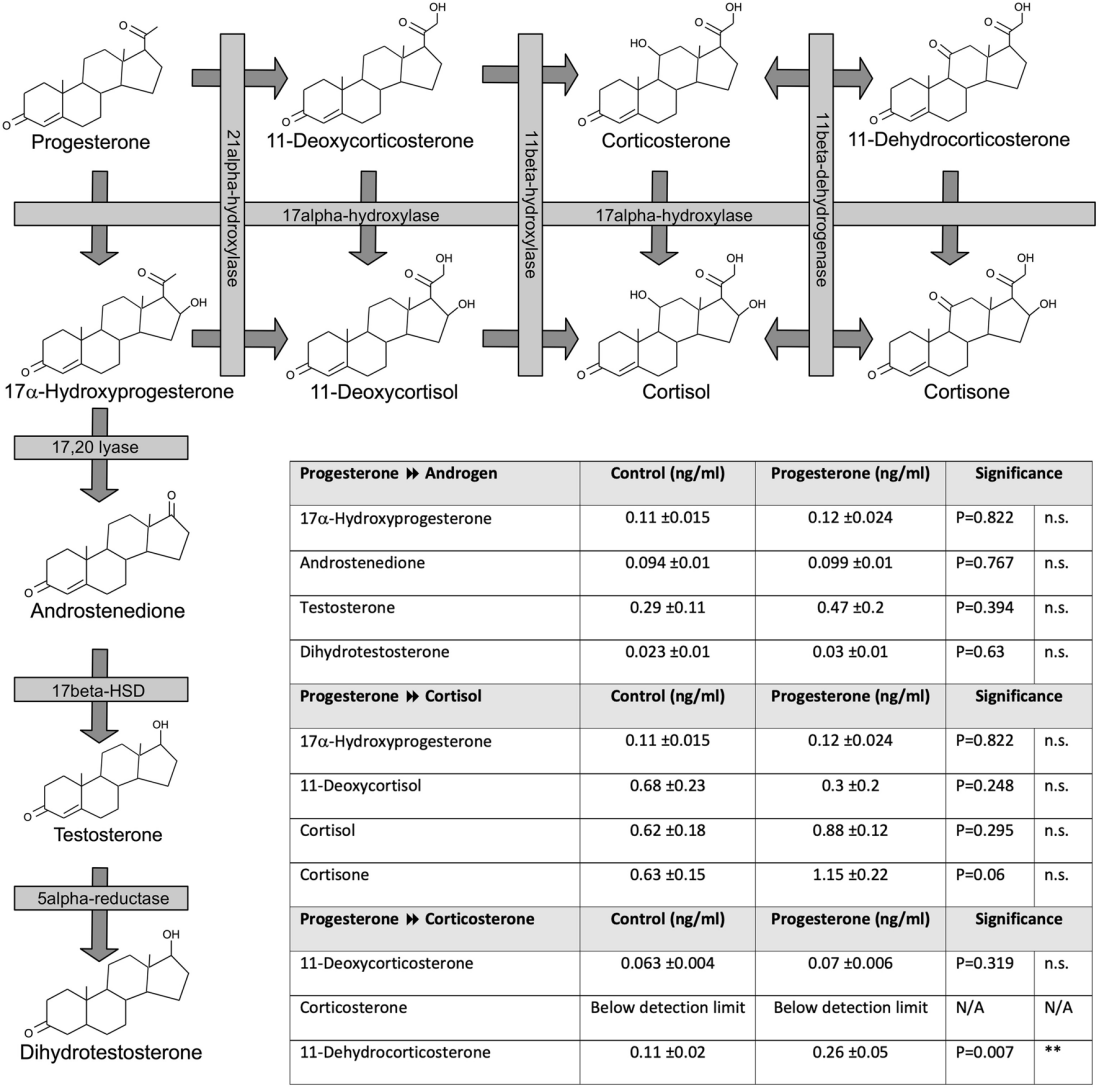
The effect of maternal progesterone administration on the metabolism of progesterone to other steroids in the male fetus at d75 gestation (C = 10, P = 7). Cartoon with chemical structures showing the enzyme pathways involved in the synthesis of androgens, cortisol and corticosterone. Maternal progesterone administration increases 11-dehydrocorticosterone concentrations in male fetuses. Unpaired, two-tailed Student’s *t* test was used for comparing means of two treatment groups with equal variances accepting *P* < 0.05 as significant (**P < 0.01).

As progesterone can be metabolised to other steroids, we assessed the steroid profile in male fetuses where the mother had been exposed in progesterone in early pregnancy. There was no effect of progesterone supplementation on the fetal androgen and cortisol pathways driven by 17α-hydroxylase (Fig. 7). However, in male fetuses there was upregulation of the progesterone to corticosterone pathway, not driven by 17α-hydroxylase, with higher concentrations of 11-Dehydrocorticosterone (Fig. 7; *P* < 0.01).

## Discussion

We investigated the fetal effects of natural progesterone administration in early pregnancy using a clinically realistic large animal model and time-frame for progesterone administration. The ovine fetus at D75 gestation is developmentally is equivalent to the 15-week human fetus. We showed that maternal progesterone administration increased progesterone concentrations only in the male fetus. This male-specific effect was also seen in female and male co-twins. We therefore focussed on the effects of this increase of fetal progesterone concentrations in the male fetuses. Investigation of the readouts of pituitary and testicular function during fetal life suggests that maternal progesterone supplementation has effects on the male fetus reproductive axis development/function in early gestation. In addition, investigating circulating hormones, where progesterone is an intermediary in their synthesis, showed an increase in 11-dehydrocorticosterone in male fetuses.

We don’t know why the male fetus increases progesterone in response to maternal administration, while the female fetus does not. There may be several explanations for this, including sexually dimorphic placental progesterone transport and metabolism. However, we chose to use in silico analysis of the fetal liver to look at potential hepatic metabolic explanations. We found sexually dimorphic expression of hepatic progesterone metabolising enzymes, with fetal males having lower expression than fetal females. This data is from D90 fetuses and so it is not clear how relevant the finding is at D75 gestation. However, it highlights that the sex differences seen in response to maternal progesterone administration is likely to be due to sexually dimorphic expression of progesterone metabolising enzymes.

We further assessed consequences of increased circulating progesterone concentrations in the male fetus by interrogating progesterone target tissues within the reproductive axis. The expression of *GNRHR*, *FSHB* and *LHB* in the male fetal pituitary was reduced after maternal progesterone supplementation. The fact that this did not occur in female fetuses, where progesterone concentrations are not different, suggests that this is a direct effect of circulating progesterone. These genes are expressed in fetal gonadotrophs and the effect was not due to a reduction in gonadotroph numbers but rather a change in function. However, we could not co-localise progesterone receptors to the gonadotrophs. Given that pituitary *PGR* expression was decreased, this suggests that effects upon *LHB* and *FSHB* accumulation are likely to be hypothalamic in origin.

Pharmacological reduction of GnRH in the fetal sheep reduced pituitary LH secretion and resulted in a 45% reduction in Sertoli cell numbers in the testis at birth^25^. This suggests a role for GnRH and gonadotrophins in in the regulation of testicular development during fetal life. Progesterone is a very potent inhibitor of GnRH neuronal activity^26^ and this postnatal feedback system might be mature and happening in the fetus. This suggests the brain itself is a target tissue for progesterone action in the male fetus. Progesterone receptors are present in the brain during fetal development and progesterone can affect neural activity^27,28^. In the late gestation, chronically catheterised fetal sheep, increasing maternal progesterone administration suppressed fetal electro-ocular activity and behavioural arousal^29,30^.

We saw effects on the developing testes. It is not clear if these are direct effects on the testes or indirect effects through suppression of gonadotrophins. We observed increased *CYP11A1* and *CYP19A1* expression in fetal testes as a consequence of increased progesterone that may be indicative of altered local steroidogenesis. There was a positive correlation between progesterone level and fetal testis expression of *STAR*, *CYP11A1* and *CYP19A1*. Aromatase (CYP19) catalyses the irreversible conversion of androgenic steroid substrates into the estrogens. Locally produced estrogens are required for normal development and fertility in males likely controlling proliferation and differentiation of Leydig, Sertoli and germ cells^31,32^. Increased testicular *CYP19A1* expression in male fetuses from early pregnancy progesterone-exposed mothers would be consistent with increased intratesticular conversion of androgens to estrogens, potentially disturbing steroidal balance.

We found that progesterone exposure is associated with an increase in 11-dehydrocorticosterone, which can act via the mineralocorticoid receptor^33,34^. It is likely that this steroid conversion takes place in the adrenal gland as 11-dehydrocorticosterone has been detected in the human fetal adrenal in the second trimester^35^. It can also be metabolised by HSD11B1 into glucocorticoids^36^. Indeed 11-Dehydrocorticosterone causes metabolic syndrome, which is prevented when *HSD11B1* is knocked out in livers of male mice^37^. It is not known what effects mineralocorticoids have in the fetus. They may have roles alongside glucocorticoids in lung development^38^ and the programming of future hypertension^39^ in animal models. It has been postulated that progesterone metabolism through corticosterone to mineralocorticoids is involved in programming autism^40,41^.

While there are changes in the pituitary and testis and circulating 11-dehydrocorticosterone we cannot determine if the fetal perturbations observed would have any long-term effects on the offspring health. We saw a similar effect on male ovine fetal pituitary function after administration of testosterone during mid-gestation^42^. While this had impacts on testicular development it normalised in later gestation after cessation of the exogenous testosterone^30^. However, in adolescence prenatally androgenised male sheep had altered testicular function as highlighted by increased AMH^13^ as well as altered spermatogenesis^43^. Changed postnatal testicular function, with increased expression of Sertoli cell *AMH* suggesting increased Sertoli cell proliferation, was also seen after prenatal progesterone treatment in mice^44,45^. It is not clear whether this would be recapitulated in the human. In the male human fetus, although there is a role for fetal pituitary function in development^46^, placental hCG is able to drive testicular function during the male programming window^47,48^.

Epidemiological evidence based on male neonatal examination suggests that progesterone supplementation in women in early pregnancy is safe. There does not seem to be an increase in congenital abnormalities in male offspring born to women after progesterone supplementation. Hypospadias was associated with maternal progesterone supplementation in cohort studies OR 3.7 (CI 2.3–6.0)^49^ when synthetic progestogen was generally used. However, an increased risk of hypospadias was not seen in the recent clinical trials using natural progesterone supplementation in recurrent miscarriage^50^ and threatened miscarriage^18^. Exposure of pregnant mice to pharmacological doses of progesterone reduced circulating testosterone levels, but does not cause abnormalities of male internal and external genitalia^51^. It is therefore likely that congenital abnormalities, which can be detected at birth, are rare after progesterone supplementation in early pregnancy and not a consequence of any effect of progesterone on the developing male reproductive system.

There is a lack of data on whether there are any longer-term functional effects of increased prenatal exposure to progesterone either on the testis or the brain in offspring and into adulthood. There are no data on the testis or pituitary and only limited data on potential brain effects in the longer term. In mice prenatal progesterone supplementation interferes with masculine behaviour in adulthood, likely due to diminished peripheral testosterone levels during the prenatal period^52^. Using a national registry of male births the duration of progesterone treatment in early pregnancy was associated with autism spectrum disorder ASD (RR 1.51: CI 1.22–1.86, *P* < 0.001)^40^. This highlights that there is the potential for postnatal effects of increased prenatal progesterone in males.

This research was done in the sheep and as such the relevance to humans is not clear. In humans and sheep progesterone is secreted by the placenta independently to the mother and the fetus, and in the human it also is much lower in the fetus than the mother^53^. In the mid-trimester human fetus no sex difference in progesterone concentrations was observed although allopregnanolone was lower in males^54^. However, in human amniotic fluid 17-OH progesterone was higher in the female fetus in the second trimester and progesterone was higher in the female fetus in the third trimester^55^.

Overall, we provide evidence that elevating progesterone in early pregnancy has acute contemporaneous effects on the developing pituitary and testes in the male fetus. We cannot say this has postnatal consequences but it is biologically plausible that it could. This means that longer term studies on male offspring exposed to increased prenatal progesterone are indicated. In IVF there is no evidence of clinical efficacy for prolonged progesterone support during pregnancy in cycles with ovarian luteal tissue^16^. In addition, there is no evidence that progesterone supplementation should be used to prevent miscarriage in women with recurrent miscarriage^50^ without bleeding or in women with bleeding in early pregnancy without previous miscarriage^18^. We should be wary of prescribing progesterone supplementation in early pregnancy outside the current evidence base in the absence of longer-term safety assessments.

## Limitations of the study

A key limitation of this study is that we have no follow up data concerning postnatal, long-term consequences. In addition, we acknowledge the lack of data on the potential contribution of the placenta or fetal weight on progesterone metabolism and clearance. Finally, we recognise that all outcomes could be measured in both sexes, however, practical constraints led us to take a data driven approach to focus on the male fetus.

## Materials and methods

### Ethics statement

All studies were approved by the UK Home Office and conducted under approved Project Licence PPL60/4401. The Animal Research Ethics Committee of The University of Edinburgh approved the study. The study was carried out in accordance with the relevant guidelines.

### Animals and tissues

Adult Scottish Greyface sheep (*Ovis aries*) typically weigh around 75 kg with intrauterine fetal developmental milestones equivalent to the human^20^. They were housed in a barn with natural light and ad libitum access to hay and water as described previously^8^. The estrous cycle was synchronised with progesterone sponges before mating with Texel rams. Pregnant ewes (n = 20) were randomised to i.m. treatment with progesterone (200 mg in vegetable oil) or vegetable oil control twice weekly from d20 of pregnancy (d147 is term). At d75 the animals were sacrificed by barbiturate overdose and maternal and fetal plasma were collected and stored at − 20 °C until analysis. Ovine fetuses at d75 of gestation have sufficiently developed gonads to examine the chosen markers of testes development and the developmental stage is approximately equivalent to the 15-week human fetus*.* Fetal tissues, (female control, n = 10; male control, n = 10; female progesterone, n = 13; male progesterone, n = 7) were collected and halved. One half of a tissue was snap frozen and stored at − 80 °C until analysis. The other half of a tissue was fixed in Bouin’s solution for 24 h and subsequently embedded into paraffin wax for subsequent immunohistochemistry.

Fetuses were from mainly from the 16 twin pregnancies (C- 3xF/F, 3xF/M, 2xM/M; P- 3xF/F, 3xF/M, 2xM/M) apart from two singletons (C- 1xM; P- 1xF) and two triplets (C- 1xF/M/M; P- 1xF/F/F). We have analysed all data with and without the non-twin pregnancies, and there was no difference to the study outcomes, either in terms of the actual results, or the significances obtained. Therefore, we have opted to keep all the fetuses in the data set.

Data from d90 fetuses was obtained from a separate study involving detailed hepatic analysis through RNA sequencing performed on control fetal female (n = 6) and male (n = 11) livers^13^. Animal husbandry, experimental protocols and tissue collection were performed exactly as previously described^56^. Ewes were sacrificed on d90 of gestation via barbiturate overdose. The rationale of d90 gestation rationale was based on that the limited available evidence demonstrated that the fetal liver expresses the majority of its systems by this stage in ovine fetuses. The gravid uterus was immediately exteriorised, fetal sex recorded and fetal hepatic tissue removed, snap frozen and stored at − 80 °C. Fetuses were from twin and singleton pregnancies, but in order to avoid any possibility of genetic bias only one animal from each pregnancy was included^56^.

### Progesterone ELISA

Serum total progesterone concentrations were determined by using a commercial Progesterone ELISA kit (Demeditec, Germany) following the manufacturer’s instructions. The minimal detectable limit level of progesterone is 0.04 ng/ml and the intra- and inter-assay co-efficients of variation were < 10%. The cross-reactivity with 11-Desoxycorticosterone was < 1.1%, Pregnenolone < 0.35%, 7αOH-Progesterone < 0.3%, Corticosterone < 0.2%, with Cortisol and DHEA-S < 0.02%, and with Estriol, Estradiol-17β, Testosterone, Cortisone and 11-Desoxycortisol < 0.01%.

### Quantitative RT-PCR

Quantitative RT-PCR was carried out using SYBR Green as described previously^42^. Primer sequences are listed in Supplementary Table 2. Real-time PCR was carried out in duplicate 10 µL reactions, and the negative controls included in each run per gene included a cDNA reaction without reverse transcriptase and a reaction replacing cDNA with nuclease-free water (template negative). The expression of the unknown target gene was analysed relative to *GAPDH* as an internal control and quantified using the ΔΔCt method. Reference gene stability was analysed via Genorm algorithm and a panel of 12 ovine reference genes (Primer Design Ltd, Southampton, United Kingdom).

### RNA sequencing transcriptomic analyses

RNA sequencing experiment was previously described in detail^13^. Libraries were prepared with the Illumina TruSeq Stranded mRNA kit, using fetal control female (n = 6) and fetal control male liver samples (n = 11) sampled at day 90 gestational age. Sequencing was performed on the NextSeq 500 High Output v2 kit (75 cycles) on the Illumina NextSeq 500 platform. To assess quality of sequencing data, reads were analysed with FastQC. To remove any lower quality and adapter sequences, TrimGalore! was used. To remove the ERCC reads, all reads were aligned to the ERCC reference genome using HISAT2. These alignments were processed using SAMtools, reads were counted using featureCounts and analysed using the R package erccdashboard. Reads were aligned to reference genome using HISAT2. SAMtools was used to process the alignments and reads were counted at gene locations using featureCounts. Pairwise gene comparisons were carried out with edgeR with all genes with CPM (count per million) value of more than one in six kept for analysis, and all other genes removed as low count genes. P values were adjusted using the Benjamini–Hochberg procedure, with a false discovery rate (FDR) set at *q* < 0.05.

### Immunohistochemistry

Immunohistochemistry was carried out as described previously^42,57^. Briefly, tissue blocks were sectioned at 5 μm thickness, mounted onto pre-labelled charged glass slides (Superfrost, Menzel GmbH & Co, Germany) and dried overnight in an oven at 50 °C. Tissue sections were dewaxed and rehydrated prior to antigen retrieval using a decloaking chamber (Biocare Medical, Concord, California) containing sodium citrate retrieval buffer (0.01 M, pH6.0). Slides were washed, incubated in 3%H_2_O_2_ for 10 min, and blocked with avidin and biotin (Vector Laboratories Ltd, Peterborough, United Kingdom) followed by 20% normal goat serum/5% BSA. Before adding the primary antibody, the tissue was blocked in normal goat serum for 30 min.

The primary antibodies (LHB – Rabbit anti-ovine LH-B [1:3,000] was supplied by Prof A.S. McNeilly: AR – Rabbit anti-human AR [1:200] (Santa Cruz biotechnology, Heidelberg, Germany)) in blocking serum were applied to tissue sections and incubated overnight at 4 °C. After washing, the sections were incubated with goat-anti-rabbit biotinylated IgG secondary antibody (Vector Laboratories Ltd, Peterborough, UK) for 1 h followed by the Vectastain ABC Elite tertiary complex (PK-1600 series; Vector Laboratories Ltd) for 1 h. Binding was visualised with 3, 3′-diaminobenzidine (Dako, Cambridge, United Kingdom). The sections were counterstained with hematoxylin and mounted. Negative controls consisted of primary antibody omission and primary antibody replaced with similar concentrations of non-specific rabbit immunoglobulins.

### Immunofluorescence

Immunofluorescence was used to colocalise PGR with LHB in the fetal pituitary. Dewaxing, rehydration, antigen retrieval, endogenous peroxidase blocking and nonspecific antigen blocking were identical to the immunohistochemistry protocol. The first primary antibody (LHB 1:10,000) in blocking serum was applied to tissue sections and incubated overnight at 4 °C. After washing, the sections were incubated with peroxidase-conjugated goat-anti-rabbit secondary antibody for 1 h followed by incubation with labelled Tyramide green (PerkinElmer Life and Analytical Sciences, Inc, Shelton, Connecticut) for 10 min. Antigen retrieval was performed before the application of the second primary antibody (rabbit anti-human PGR [1:300] (Santa Cruz Biotechnology)), and binding was detected using Tyramide red (PerkinElmer Life and Analytical Sciences, Inc, Shelton, Connecticut). Slides were mounted using Permafluor (Immunotech, Marseille, France) and images captured using the LSM 710 Confocal microscope (Carl Zeiss, Hertfordshire, United Kingdom).

### LC–MS/MS

Steroid quantities in the plasma samples were obtained following extraction and LC–MS/MS analysis^58^. Briefly calibration curves were prepared alongside fetal plasma samples (200 µl) enriched with isotopically labelled internal standards. These were extracted using Supported Liquid Extraction SLE400 cartridges (Biotage, UK) by diluting in 0.5 M ammonium hydroxide (200 µl), loading, eluting with (95:5) dichloromethane/isopropanol (0.45 ml × 3), drying under nitrogen and resuspending in 70:30 water/methanol. Chromatographic separation was achieved using a gradient on a Shimadzu Nexera on a Kinetex C18 (150 × 3 mm; 2 µm) column and mobile phases: A- 0.1% FA in water, B –0.1% FA in methanol, 0.5 ml/min, 30C, followed by MS analysis on a Sciex QTrap 6500+ operated in positive ESI with scheduled MRM to maximise sensitivity.

Steroids were correctly identified according to their retention time, from known calibration standards, and parent-product mass transitions. Steroids quantified were cortisol, cortisone, 11-deoxycortisol, 11-deoxycorticosterone, corticosterone, 11-dehydrocorticosterone, androstenedione, dihydrotestosterone, testosterone, with calibration curves ranging between 0.0025 and 10 ng. Least squares regression of the peak area ratio, with 1/x weighting, was used to calculate the amount of steroid in each sample in Analyst MultiQuant software (Sciex, UK). EMA bioanalytical method validation guidelines were used to establish limits of detection, assay precision and accuracy for each steroid of interest.

### Statistical analysis

Whole pituitary mid-section LHB area of staining was examined blindly and quantified using Image J analysis (https://imagej.nih.gov) with fixed intensity thresholding. In cases of single gene analyses, all data sets were normality tested prior to further analysis (Shapiro–Wilk test), and logarithmically transformed if necessary. For comparing means of two treatment groups with equal variances, unpaired, two-tailed Student’s *t* test was used accepting *P* < 0.05 as significant. Maternal progesterone was analysed using unpaired, one-tailed Student’s *t* test. The effect of progesterone treatment on female and male fetuses was analysed using two-way ANOVA with Tukey post hoc test. Co-twin fetal serum progesterone level between male and female was analysed using a paired two-tailed Student’s *t* test and between progesterone and control a one-tail unpaired Student *t* test. Correlation was assessed by calculation of Pearson product-moment co-efficient. Statistical analysis was performed using GraphPad Prism 8.0 software (GraphPad Prism Software, San Diego, CA, USA). Asterisks were used to indicate level of significance based on the following criteria: *P < 0.05, **P < 0.01, ***P < 0.001 and ****P < 0.0001.

## Supplementary information

Supplementary Figure 1.

Supplementary Tables.
