# Supplementary material for: Early pregnancy maternal progesterone administration alters pituitary and testis function and steroid profile in male fetuses
Source: Sci Rep. 2020 Dec 14;10:21920. doi: 10.1038/s41598-020-78976-x (PMC7736841; doi:10.1038/s41598-020-78976-x)
Supplement: Supplementary file 1 — Supplementary Figure 1. [file 41598_2020_78976_MOESM1_ESM.pdf]

Early pregnancy maternal progesterone administration alters pituitary and testis function and steroid profile in male fetuses

Authors: Katarzyna J. Siemienowicz, Yili Wang, Magda Marečková, Junko Nio-Kobayashi, Paul A. Fowler, Mick T. Rae, W. Colin Duncan

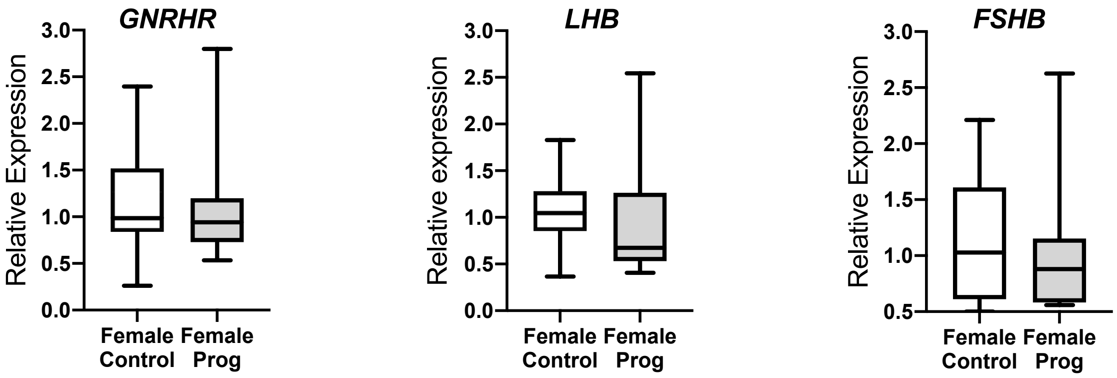

Supplementary Figure 1. The effect of maternal progesterone on pituitary gene expression in female fetuses at d75 gestation compared to vehicle controls. Maternal progesterone administration had no effect on the expression of *GNRHR*, *FSHB* and *LHB* in fetal females (C=10; P=13). Box plot whiskers are lowest and highest observed values, box is the upper and lower quartile, with median represented by line in box. Unpaired, two-tailed Student's t test was used for comparing means of two treatment groups with equal variances accepting $P<0.05$ as significant.
