# Supplementary material for: Early pregnancy maternal progesterone administration alters pituitary and testis function and steroid profile in male fetuses
Source: Sci Rep. 2020 Dec 14;10:21920. doi: 10.1038/s41598-020-78976-x (PMC7736841; doi:10.1038/s41598-020-78976-x)
Supplement: Supplementary file 2 — Supplementary Tables. [file 41598_2020_78976_MOESM2_ESM.pdf]

Early pregnancy maternal progesterone administration alters pituitary and testis function and steroid profile in male fetuses

Authors: Katarzyna J. Siemienowicz, Yili Wang, Magda Marečková, Junko Nio-Kobayashi, Paul A. Fowler, Mick T. Rae, W. Colin Duncan

Supplementary Table 1

Pituitary		
**Gene**	**Pearson r**	**P value**
PR	-0.55	0.017
FSHB	-0.59	0.016
LHB	-0.51	0.046
**Testis**		
**Gene**	**Pearson r**	**P value**
POU5F1	0.54	0.038
LHR	0.67	0.006
STAR	0.53	0.049
CYP11	0.69	0.004
CYP17	0.51	0.049
CYP19	0.86	<0.0001
AMH	0.51	0.049
SHBG	0.62	0.014

Correlation of genes in pituitary and testes with progesterone levels in fetal males. Correlation was assessed by calculation of the Pearson r product-moment co-efficient, with $P<0.05$ accepted as significant.

Supplementary Table 2

Gene	Forward Primer	Reverse Primer
PGR	CCCTAGCTCACAGCGTTTCT	CCCGGGACTGGATAAATGT
GAPDH	GGCGTGAACCACGAGAAGTATAA	AAGCAGGGATGATGTTCTGG
GNRHR	GCAGTGAAAAGCAACAGCAA	GGCAGCTGAAGGTGAAAAAG
LHB	TCACTTTCACCACCAGCATC	AAGGAGACCATTGGGTCCAC
FSHB	TGTTGCTGGAGAGCAATCTG	AGTCTGCATGGTGAGCACAG
LHR	TCCGAAAGCTTCCAGATGTT	GAAATCAGCGTTGTCCCATT
STAR	GCATCCTCAAAGAACAGGAG	CTTGACACTGGGGTTCCACT
CYP11	CAACGTCCCTCCAGAACTGT	CAGGAGGCAGTAGAGGATGC
HSD3B1	GGAGACATTCTGGATGAGCAG	TCTATGGTGCTGGTGTGGA
CYP17	AGACATATTCCCTGCGCTGA	GCAGCTTTGAATCCTGCTCT
INSL3	ATGGACCGTCGTCCGCTC	TCAGTGGGGACAGAGGGTCAG
POU5F1	CGAAGCTGGACAAGGAGAAG	AGAGAACCCCCAGGGTGAG
VASA	TATATGGGGGAACCCAGTTG	CATATCCCAGCATGCGATCAG
DMRT1	TCGTTTCCGACTCCACCTAC	CCTGTCTGACCAGGCACATA
WT1	AGCAACCAAGGAGCAAGAAA	GCGTAAACTTCCAGGCACTC
SOX9	CTCAAGGGCTACGACTGG	GTGCAGGTGCGGGTACTG
FSHR	TAAGCACTTGCCAGCTGTTC	CTCATCGAGTTGGGTTCCAT
AMH	GGCCTGAGTGACTTGACCAC	CCTGGGTACAACACCAGCAG
SHBG	TCACTGGGCTCAGCTTACAGT	CCACCCTGAGTAGCAAGGAA
CYP19	AATCCAGCACTCTGGAAAGC	ACGTCCACATAGCCCAAGTC

Primers for real-time RT-PCR analysis. Forward (F) and reverse (R) primers (Table 1) were designed using Primer3 Input version 0.4 online software (http://frodo.wi.mit.edu) with DNA sequences obtained at Ensembl Genome Browser. To confirm the validity of the gene product in the sheep, both conventional PCR and amplicon sequencing were performed. Primer specificity and efficacy for qRT-PCR was evaluated through the generation of standard curves with serial dilutions of cDNA; a standard curve slope of approximately -3.3 was accepted as efficient, and a melt-curve analysis was also performed.
